# Distribution of macroalgae in the area of Calvi (Corsica)

**DOI:** 10.3897/BDJ.9.e68249

**Published:** 2021-08-23

**Authors:** Lea Katz, Damien Sirjacobs, Sylvie Gobert, Pierre Lejeune, Bruno Danis

**Affiliations:** 1 Université Libre de Bruxelles, Bruxelles, Belgium Université Libre de Bruxelles Bruxelles Belgium; 2 Université de Liège, Liège, Belgium Université de Liège Liège Belgium; 3 STARESO, Calvi, France STARESO Calvi France

**Keywords:** macroalgae, ROV, Calvi, *
Cystoseira
*, *
Caulerpa
*, benthic habitat

## Abstract

**Background:**

Macroalgae play a structuring role in benthic ecosystems, which makes it very important to monitor their cover rates and study their community structures and changes in time. Such studies are usually led by autonomous divers and often do not generate sufficient data to provide enough material for strategically-sound conservation plans. This paper describes the dataset generated in the framework of the evaluation of the potential of a complementary data acquisition method: annotating videos transects obtained using an underwater Remotely Operated Vehicle (ROV).

The focus was on *Cystoseirabrachycarpa*, together with the abundance of several other macroalgae species, which could be identified using the ROV images after validation through specimen identification. Furthermore, in order to allow future uses, such as monitoring the status of colonisation of the invasive algae *Caulerpacylindracea*, the ROV was sent to some deeper transects on sedimentary habitats (40 m) below the *Posidonia* meadows lower limits.

**New information:**

The project, while providing some interesting insights on using a ROV as a tool to study marine biodiversity, generated a dataset for the distribution of 19 macroalgae on both standardised and new transects in the Bay of Calvi (Katz et al. 2021). The observed species of macroalgae included: *Acetabulariaacetabulum*, *Amphiroarigida*, *Caulerpaprolifera*, *Caulerpacylindracea*, *Codiumbursa*, *Colpomeniasinuosa*, Corallinales (order), *Cystoseirabrachycarpa*, *Cystoseiracrinita*, *Cystoseiraspinosa*, *Cystoseirazosteroides*, *Dictyota* (genus), *Flabelliapetiolata*, *Halopteris* (genus), *Halopterisscoparia*, *Jania* (genus), *Osmundariavolubilis*, *Padina pavonica* and *Peyssonneliasquamaria*.

The videos also showed that the invasive algae *Caulerpacylindracea* has spread between 2016 and 2019 and that more focused studies should be held in the Bay to assess the actual reach and possible impacts of this invasion.

Finally, our ROV video transects have also underlined the significant presence of particular benthic macroalgae communities over habitat zones described as "soft-bottom" on benthic habitat maps. Although the biomass per unit area of these communities is probably lower than for most coastal rocky bottoms, this widely-spread habitat type holds a contribution to primary production to be considered in coastal ecosystem models.

## Introduction

As macroalgae play an essential role in benthic ecosystems, it can be very important to monitor their cover rates and study their community structures. A good knowledge on algae distributions of an area can tell us a lot about the dynamics of the ecosystem, its resilience and the pollution in the area. For example, the analysis of the cover rates of different macroalgae, often used as bioindicators of environmental disturbance, in particular the genus *Cystoseira*, allows us to assess the impacts and pressures of environmental changes in the Bay of Calvi. Such approaches already exist for *Cystoseira* in the Mediterranean (Sales Villalonga et al. 2010), as for complete shallow macroalgae communities (Leduc et al. 2017, Sirjacobs et al. 2019).

There are several approaches to studying macroalgae distributions. The most common method is by autonomous divers. Diver-based surveys are relatively simple to carry out and generate data with a high level of precision. However, this method has some caveats: there is a need for highly experienced autonomous divers, it can be destructive and is limited in time and depth and, thus, can only be used for small scale studies. Using a Remotely Operated Vehicle, equipped with cameras filming the bottom, can address many of these problems. It not only optimises the sampling design, but also generates much more data in less time.

In this project, the distribution of 19 macroalgae were recorded, but the focus was put on the species *Cystoseirabrachycarpa* and *Caulerpacylindracea*. The *Cystoseira* “forests” represent the largest biomass in the rocky bottoms of the Mediterranean and give the seascape a tri-dimensional structure, which makes them key species for the ecosystem because they serve as a habitat for many benthic organisms (Robvieux 2013). The most common species of this genus in the Bay of Calvi is *Cystoseirabrachycarpa* (Hoffmann et al. 1992). This perennial species is particularly vulnerable to environmental changes because of its low dissemination capacity and weak recolonisation rate (Sales Villalonga et al. 2010). Other species of *Cystoseira* can also be observed in the area (Coppejans and Boudouresque 1983), but are less abundant in the studied depth range (0-40 m): Cystoseiraamentaceavar.stricta, *Cystoseiracompressa*, *Cystoseiracrinita*, *Cystoseiraspinosa*, *Cystoseirazosteroides* and *Cystoseirafoeniculaceae*.

*Caulerpacylindracea* is an invasive algae originating from southwest Australia. Along with *Caulerpataxifolia*, another invasive species of the same genus, it has invaded many of the coastal habitats of the Mediterranean Sea (Piazzi et al. 2005). When *Caulerpacylindracea* is present in an ecosystem, it competes with native algae by covering them with its multi-layer stolon mats and, thus, reduces their cover rate and diversity. Furthermore, if highly abundant, it can reduce biomass and density of *Posidonia* meadows (Lamare and Verlaque 2017). Close monitoring of its dispersion is therefore necessary (Cariou et al. 2014).

### Area of Interest

**Figure 1.**
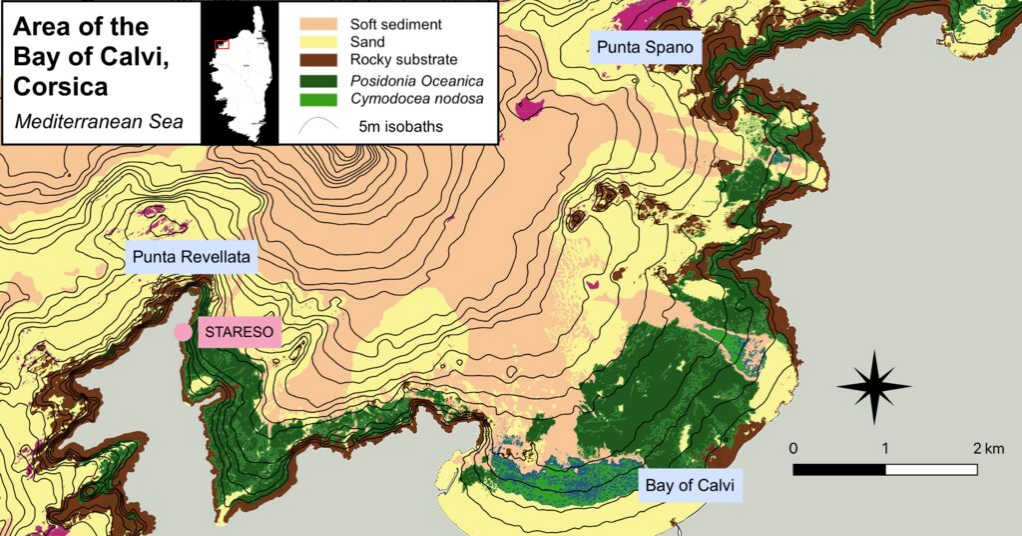
Map of the studied area.

Located in the northwest of Corsica (Fig. 1), the Bay of Calvi is an obvious choice as a study site because not only does it harbour STARESO, a research station that has over 40 years of data on macroalgae distributions and other physical parameters of the region (Coppejans and Boudouresque 1983,Demoulin et al. 1980,Janssens et al. 1993), it also acts as a reference for the study of Mediterranean coastal communities because it harbours all types of coastal habitats of the Mediterranean Sea (Andral et al. 2006), while at the same time being relatively unpolluted. However, due to its evergrowing local tourism and on-going global climate changes, monitoring and conservation actions should be taken in the near future to prevent the degradation of this pristine area.

### Ecological context and perspectives

In the Mediterranean, the most diverse benthic communities are found in the coastal areas, but these areas are also amongst the most exposed to fishing, as well as disturbances arising from urbanisation and industrialisation of the coast as corollary expansions of infrastructure for expanding tourism activities. Calvi, the city located in the centre of our area of interest, sees its population increasing 60-fold every summer (more than 360,000 inhabitants, INSEE 2017). This increase has many impacts on the surrounding coastal environment. Noise coming from boats (both for transportation and leisure (Magnier et al. 2016), damage to *Posidonia* meadows by anchoring (Abadie et al. 2017) and trampling by divers are just a few examples.

**Figure 2.**
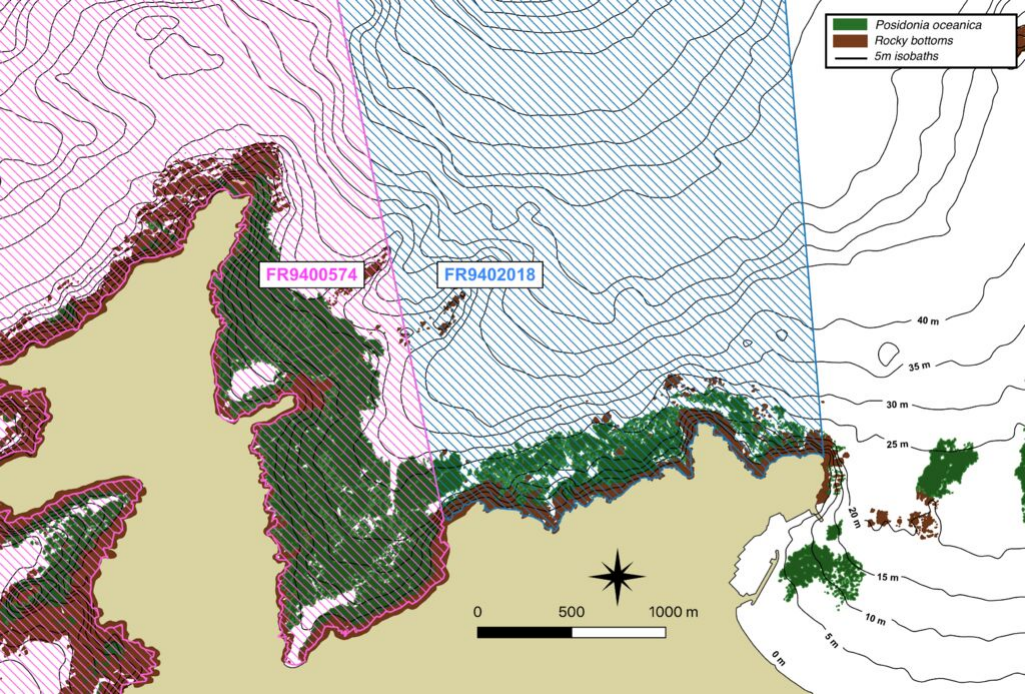
Protected areas in the Bay of Calvi as part of the Natura 2000 network. In pink, "FR9400574" (est. 2002). In blue, "FR940218" (est. 2008).

Two Natura 2000 areas from the "Habitats Directive" network are present in the Bay of Calvi (Fig. 2). These were established in 2002 and 2008, respectively and were mainly designated to protect the *Posidonia* meadows (*Posidoniaoceanica* (L.), Delile 1813, an endemic phanerogam of the Mediterranean), coralligenous reefs and underwater caves, as habitats for a richly diverse fauna and flora (FR9400574 and FR9402018, *EEA 2019*). These two sites cover a total of 124,366 ha. Apart from these two sites, no other conservation measures are being taken in the Bay of Calvi.

The dataset describes the recent state of macroalgae distributions in the area of Calvi and suggests a new sampling method that could benefit ongoing monitoring actions. The sampling campaigns were designed to follow-up on previous algae surveys in the area and were carried out on the same standardised transects. Therefore, the data we provide can be used to study the evolution of macroalgae distributions on a 40 year-span (since 1979, Demoulin et al. 1980, Hoffmann et al. 1988, Janssens et al. 1993,Sirjacobs et al. 2019) and to explore additional ones, including in sedimentary habitats. This could give interesting insights on how water quality improved after installation of a dedicated plant to treat wastewater discharges in the Bay of Calvi (Clarisse 1984, Janssens et al. 1993) and how this affected benthic communities. Moreover, these data and future sampling events following the same protocol could be used to assess the usefulness of the Natura 2000 initiatives, as well as the possible impacts of the *Caulerpacylindracea* invasion.

## Project description

### Title

Evaluation of ROV imagery for the study of macroalgae communities in the Bay of Calvi, Corsica. Masters Thesis

### Personnel

This project was driven by Lea Katz's Masters Thesis, co-supervised by Damien Sirjacobs, Sylvie Gobert (Université de Liège) and Bruno Danis (Université Libre de Bruxelles). NB: First sampling campaign in 2016 was led by Antonio Aguera Garcia (Université Libre de Bruxelles) and Damien Sirjacobs.

### Study area description

The area of interest was the near-shore area of the Bay of Calvi (Fig. 1). In particular, the rocky bottoms, the areas around *Posidonia* meadows and a few bare sediment areas were studied. The analysed area covered the 0-40 m depth layer.

### Funding

This Masters Thesis was organised and funded by the Marine Biology Lab of the Université Libre de Bruxelles. The project received logistics support from the STARESO station, Université de Liège (Pierre Lejeune). The project was also part of the STARE-CAPMED framework (STAtion of Reference and rEsearch on Change of local and global Anthropogenic Pressures on Mediterranean Ecosystems Drifts), which aims to "improve the understanding of how different coastal marine ecosystems function in the Mediterranean and describe the influence of local and global pressures on the processes that govern its functioning" (Leduc et al. 2017).

## Sampling methods

### Study extent

**Figure 3.**
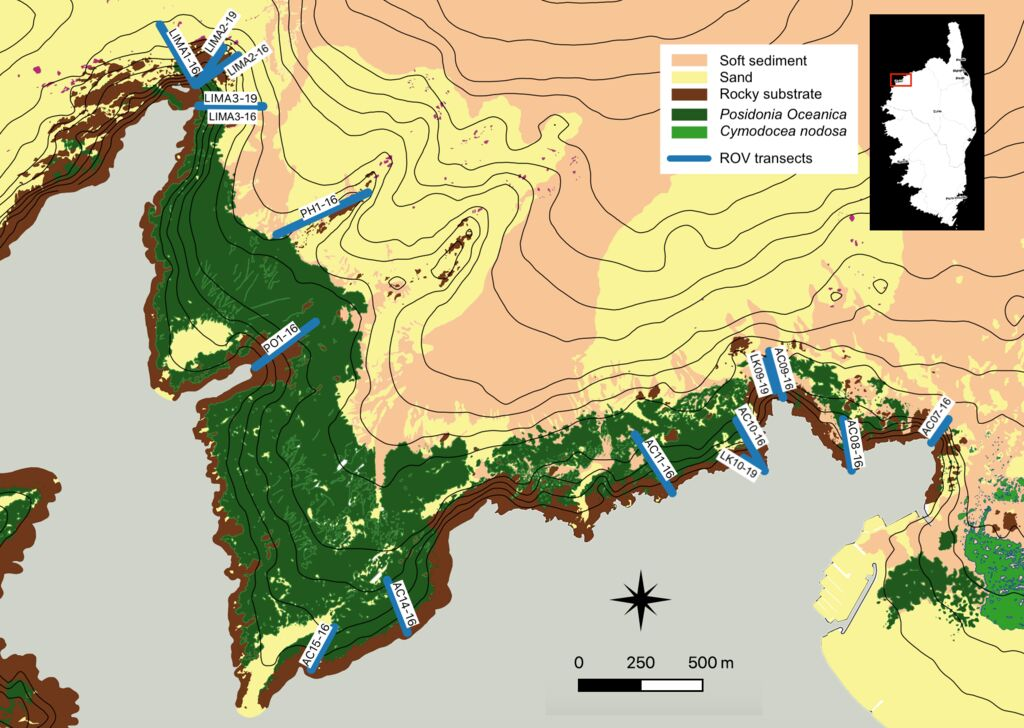
Map of annotated ROV transects.

The project included two sampling campaigns. Both campaigns covered the same geographic area (see "geographic coverage"). The underwater ROV was guided over several standardised transects to film the bottom (Fig. 3). From one year to another, some transect locations were identical, while a few others were slightly different. The length of the transects was variable: around 100 m in general, but reaching 500 m for the longest ones in deep habitats or low-pitched slope. The first campaign occurred in July 2016 and was performed over several days. Each transect was observed once. The second campaign took place in June 2019. Again, several days were needed to observe the different transects. Each transect was observed once.

### Sampling description

In both campaigns (2016 and 2019), an underwater Remotely Operated Vehicle (ROV) was deployed in the Bay and navigated along straight transects perpendicular to the coastline. To choose the location of our transects, we considered previously-studied transects in older studies that focused on the same macroalgae communities (Demoulin et al. 1980, Janssens et al. 1993). Several additional transects were also studied to improve knowledge of deeper parts of the Bay (that are usually not studied by divers) and to study the extent of the colonisation of *Caulerpacylindracea*. Although both campaigns were similar in design, there were some differences in the equipment.

**Figure 4.**
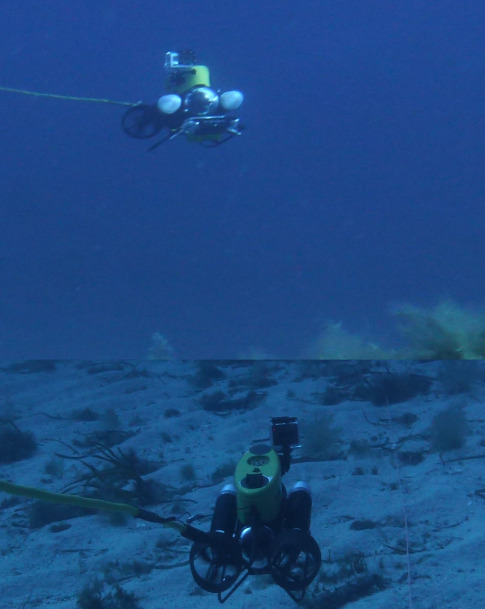
Underwater view of the mini-ROV VideoRay Pro 3. Picture : Sirjacobs et al. 2017

**Figure 5.**
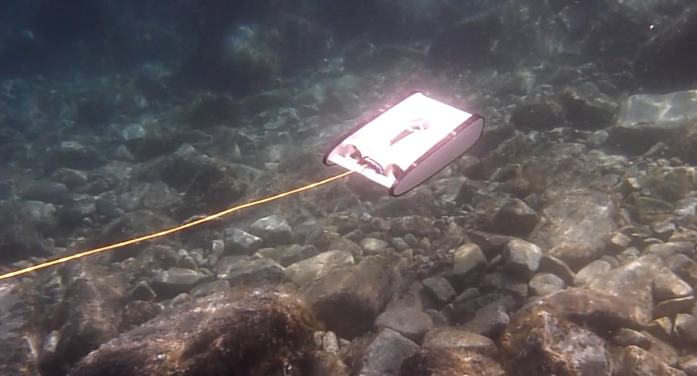
Underwater view of the OpenROV Trident.

**Figure 6.**
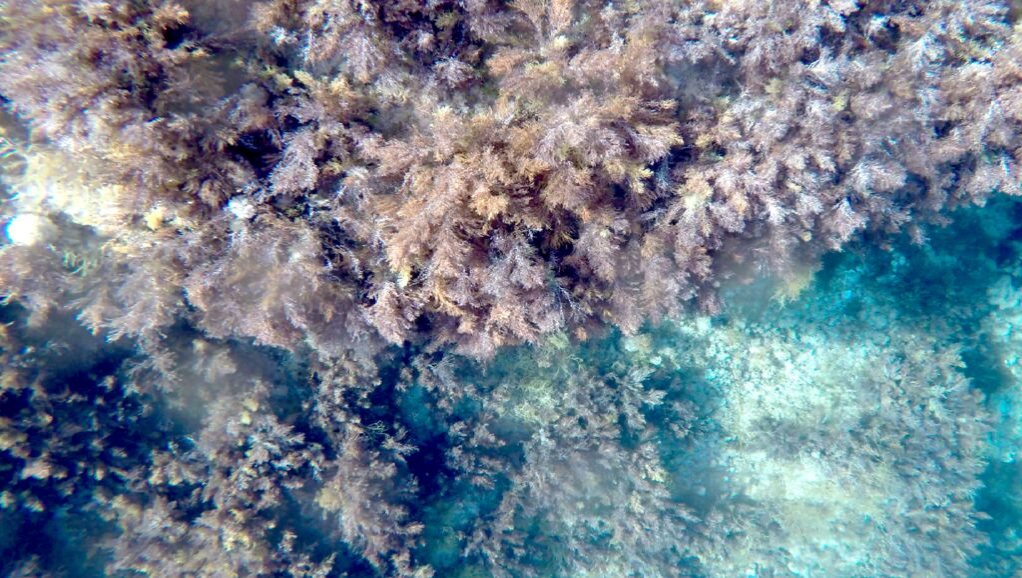
*Cystoseira* forests in the Bay of Calvi (2019). View from down-looking GoPro camera attached to a ROV.

**Figure 7.**
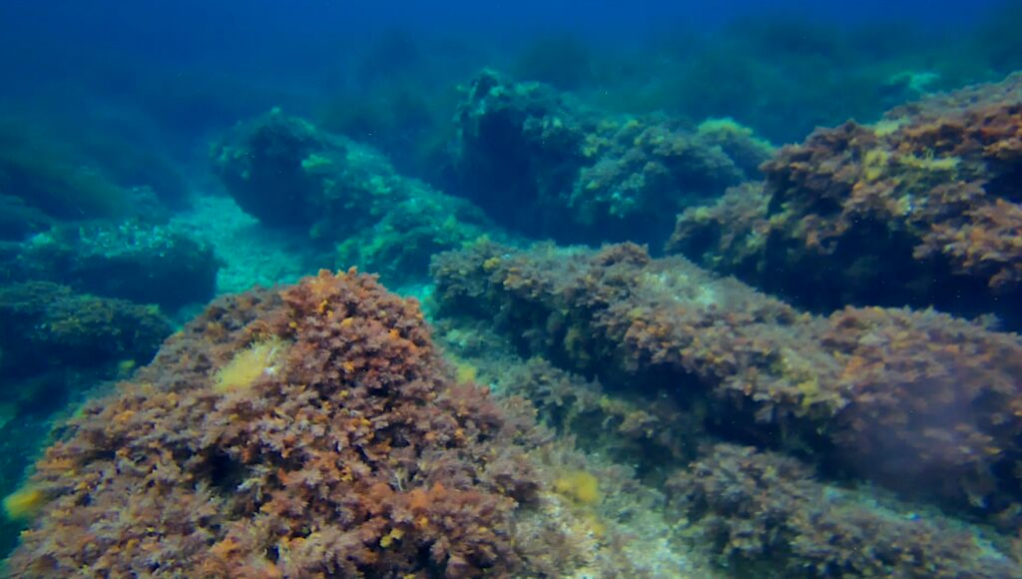
Rocky bottoms with *Cystoseira* forests in the Bay of Calvi (2019). Front-looking view from built-in camera on the ROV.

In the 2016 campaign, a mini-ROV VideoRay Pro 3 (Fig. 4) was equipped with 3 GoPro Hero3 cameras for recording the videos (two down-looking, one front-looking) in addition to the built-in camera which was used for navigation only. The mini-ROV was also equipped with a small CTD device to record salinity, temperature and depth. In the 2019 campaign, the ROV was an OpenROV Trident (Fig. 5). It was equipped with a GoPro Hero4 camera (down-looking, for video recording) along the built-in camera of the ROV (front-looking, for navigation and recording). Temperature and depth were recorded by a built-in sensor. In both cases, algae were identified and their coverage was quantified using the down-looking videos (Figs 6, 7).

Videos were visualised and annotated using the software "COVER" (Carré 2010). For each transect, species were identified and their relative abundance was estimated (as cover rate). All species identifications were made by one observer (Lea Katz), who received extensive training on algae identification by an expert at ULiège and STARESO (Damien Sirjacobs). Samples were taken back to the lab to help with some identifications. Overall, most common algae species were easily identified on the videos.

### Quality control

One transect was replicated by a team of divers who carried out independent identifications. This proved that our ROV/video survey method was robust and provided similar results for main taxon levels (cfr. "Taxonomic coverage").

## Geographic coverage

### Description

The area of interest is the Bay of Calvi (Corsica, Mediterranean Sea). Total coverage of the dataset is the extent of 17 ROV transects (12 carried out in 2016, five in 2019), perpendicular to the coastline. Transects are distributed in the whole Bay of Calvi, Corsica (Mediterranean Sea). More specifically, the area between Punta Revellata and Punta Spano was the focus of our study (Fig. 1).

### Coordinates

42.553 and 42.617 Latitude; 8.709 and 8.83 Longitude.

## Taxonomic coverage

### Taxa included

**Table:** 

Rank	Scientific Name	
species	* Acetabularia acetabulum *	
species	* Amphiroa rigida *	
species	* Caulerpa prolifera *	
species	* Caulerpa cylindracea *	
species	* Codium bursa *	
species	* Colpomenia sinuosa *	
species	* Cystoseira brachycarpa *	
species	* Cystoseira crinita *	
species	* Cystoseira spinosa *	
species	* Cystoseira zosteroides *	
species	* Flabellia petiolata *	
species	* Halopteris scoparia *	
species	* Osmundaria volubilis *	
species	*Padina pavonica*	
species	* Peyssonnelia squamaria *	
genus	*Dictyota* sp.	
genus	*Halopteris* sp.	
genus	*Jania* sp.	
order	Corallinales	

## Temporal coverage

**Data range:** 2016-7-17 – 2016-7-21; 2019-6-05 – 2019-6-07.

### Notes

Two sampling campaigns were held, one in July 2016 and one in June 2019.

## Usage licence

### Usage licence

Creative Commons Public Domain Waiver (CC-Zero)

## Data resources

### Data package title

Distribution of Macroalgae in the area of Calvi (Corsica, Mediterranean Sea)

### Resource link

https://www.gbif.org/dataset/e82beebb-d3ce-4236-a46f-aec303d1cc86#

### Number of data sets

1

### Data set 1.

#### Data set name

Distribution of Macroalgae in the area of Calvi (Corsica, Mediterranean Sea)

#### Data format

DarwinCore Archive

#### Number of columns

27

#### Description

This is the dataset associated with the datapaper. Each transect that was studied is referenced geographically. The dataset represents the presence of the algae species on the transects.

**Data set 1.** 

Column label	Column description
occurrenceID	ID label of the occurrence
eventID	ID label of the event (transect)
basisOfRecord	What the record is based on. Only "HumanObservation" in this case.
type	Type of observation. Only "MovingImage" in this case, since the observations were made from videos.
recordedBy	Name of the observer. Here : Lea Katz
kingdom	Kingdom of the observed specimen
scientificName	Scientific name of the observed specimen
taxonRank	Taxon rank of the observed specimen
eventID	ID label of the event (transect)
InstitutionCode	Code name of the institution. Here : ULB (Université Libre de Bruxelles)
eventDate	Date of the event
year	Year of the event
month	Month of the event
day	Day of the event
decimalLongitude	Longitude coordinate of the mid-point of the transect
decimalLatitude	Latitude coordinate of the mid-point of the transect
footprintWKT	Well-known text representation of the geometry of the sampling event. Here : "LINESTRING" because the transects are linear.
maximumDepthInMetres	Maximum depth of the transect in metres
waterBody	Water body where the sampling took place. Here: Mediterranean Sea
higherGeography	Geographic region where the sampling occurred
island	Island around which the sampling occured. Here: Corsica
countryCode	Code of the country where the sampling occurred. Here: "FR" (France)
GeodeticDatum	Spatial reference system upon which the geographic coordinates given in decimalLatitude and decimalLongitude are based. Here: WGS84 (World Geodetic Datum 1984)
CoordinateUncertaintyInMetres	Uncertainty of the GPS coordinates. In metres.
SampleSizeValue	Length of the transect
SampleSizeUnit	Unit of the "SampleSizeValue". Here: "metres"
samplingProtocol	Protocol used for the sampling event. Here: "Remotely Operated Vehicle"
